# Obesity Has an Interactive Effect with Genetic Variation in the Activating Transcription Factor 6 Gene on the Risk of Pre-Diabetes in Individuals of Chinese Han Descent

**DOI:** 10.1371/journal.pone.0109805

**Published:** 2014-10-10

**Authors:** Nan Gu, Xiaowei Ma, Junqing Zhang, Aimei Dong, Mengmeng Jin, Nan Feng, Hong Zhang, Xiaohui Guo

**Affiliations:** Endocrinology Department, First Hospital, Peking University, Beijing, China; Virgen Macarena University Hospital, School of Medicine, University of Seville, Spain

## Abstract

Endoplasmic reticulum (ER) stress is one of the contributing factors to the development of β-cell failure in type 2 diabetes. ER stress response through ATF6 has been shown to play an important role in insulin resistance and pancreatic β-cell function. We investigated whether genetic polymorphisms in ATF6 were associated with the risk of pre-diabetes in a Chinese Han population, and whether they had a synergistic effect with obesity. Our samples included 828 individuals who were diagnosed as pre-diabetic, and 620 controls. The minor allele A at rs2340721 was associated with increased risk for pre-diabetes(*p* = 0.013), and this association was still significant after adjusting for gender, age, body mass index (BMI), and waist-hip ratio(*p*′ = 0.011). BMI, treated as a continuous variable, and rs2340721 had an interactive effect on pre-diabetic risk(*p* for interaction = 0.003, β = 0.106). Carriers of GG at rs7522210 were also at a higher risk compared to non-carriers (OR = 1.390, 95%CI:1.206–1.818, *p* = 0.013, adjusted OR′ = 1.516, 95%CI:1.101–2.006, *p*′ = 0.006). GG homozygotes had increased fasting blood glucose (FBG) levels(GG vs CX: 5.6±0.52 vs 5.5±0.57 mmol/L, *p* = 0.016), lower insulin levels (0,30,120 minutes after glucose load) (p<0.05), and reduced areas under the insulin curve than non-carriers(GG vs CX:67.3(44.2–102.3) vs 73.1(49.4–111.4), *p* = 0.014). rs10918270 was associated with FBG, and rs4657103 with 2 hour glucose levels after a 75 g glucose load. We also identified a haplotype of TTAG composed of rs4657103, rs2134697, rs2340721, and rs12079579, which was associated with pre-diabetes. The genetic variation in ATF6 is associated with pre-diabetes and has interactive effects with BMI on pre-diabetes in the Chinese Han population.

## Introduction

Prevalence of type 2 diabetes has been rising rapidly, and it has been a major public health challenge worldwide, especially in China. Pancreatic β-cell dysfunction is a key causal factor in the development and progression of type 2 diabetes. In the early stage of the disease, pre-diabetes, characterized by impaired fasting glucose (IFG) and/or impaired glucose tolerance (IGT) [1], pancreatic β-cell dysfunction and insulin resistance have already presented. Several genetic and environmental factors are likely to contribute to the progressive abnormality or even failure of pancreatic β-cell function, but the precise mechanism is still unknown. Endoplasmic reticulum (ER) stress is thought to be a contributing factor. The unfolded protein response (UPR), induced by the accumulation of unfolded or misfolded proteins in the ER, plays an important role in chronic metabolic diseases, such as insulin resistance and type 2 diabetes. ATF6 is an ER stress-regulated transmembrane transcription factor in the UPR signaling pathway that activates the transcription of ER molecular chaperons to alleviate ER stress. We inferred that the genetic variability in ATF6 may contribute to the changes in pancreatic β-cell function or insulin sensitivity. To test this hypothesis, we studied the genetic variations in ATF6 in a sample of pre-diabetic Chinese individuals.

## Materials and Methods

### Ethics statement

The study protocol and informed written consent forms were approved by the research ethics committee of Peking University First Hospital, and the study was carried out in compliance with the Helsinki Declaration. All patients signed an informed written consent form to participate in the study.

### Study population

Subjects were recruited from the database for the Epidemiological Survey on Diabetes that was conducted in three large communities in Beijing from 2007 to 2009. A total of 1,448 non-consanguineous subjects with complete medical records were enrolled in the study. Based on the oral glucose tolerance test (75 g OGTT), and according to the 1999 WHO diagnostic criteria for diabetes and pre-diabetes, 828 subjects were confirmed pre-diabetic (defined as fasting glucose [FBG] between 6.1 mmol/L [100 mg/dL] and 7.0 mmol/L [126 mg/dL], or 2 hours post-OGTT glucose [PBG] between 7.8 mmol/L [140 mg/dL] and 11.1 mmol/L [200 mg/dL], or both). Another 620 healthy individuals who had no family history of diabetes and were ≥55 years of age served as controls.

### Methods

All subjects underwent a standardized clinical and laboratory evaluation. A standard questionnaire for epidemiological survey was used, and the items included general information, family history, medical history, and medication. During the evaluation, body height, body weight, waist circumference, hip circumference, and blood pressure were measured. In the morning, fasting blood was collected for the measurement of glucose and lipid levels (Table 1). The remaining 2–4 ml of venous blood was used for DNA extraction using the salting-out method [20]. Homeostasis model assessments of insulin resistance (HOMA-IR) and β-cell function (HOMA-β) were calculated using the formula described by Matthews et al. [2]. Fasting and 2 hours PBG were measured enzymatically. The coefficient of variation was <2.5% and inter-assay coefficient of variation was <3.5%. Total cholesterol (TC), triglycerides (TG), and high-density lipoprotein cholesterol (HDL-C) were measured using the Hitachi 7600 automatic biochemical analyzer with the coefficient of variation of <1.5% and inter-assay coefficient of variation of <3.0%. Fasting insulin levels (INS0), post-OGTT30′ insulin levels (INS30) and 2 hour post-OGTT insulin levels (INS120) were measured by chemiluminescence using Roche Cobas e 601 Analyzer, while the coefficient of variation was 1.8%–2.0%, and the inter-assay coefficient of variation was 4.7%–7.9%. Homeostasis model assessments of insulin resistance (HOMA-IR), β-cell function (HOMA-β), and areas under the curve for insulin (AUCi) were calculated using the formula [HOMA-IR  =  fasting insulin × fasting plasma glucose/22.5, and HOMA-β = 20× fasting insulin/(fasting plasma glucose - 3.5)] described by Matthews et al. [15].

**Table 1:** Clinical characteristics of pre-diabetic cases and controls in China.

	Controls	p-DM	
	(n = 621)	(n = 845)	*P-*value
Gender (M/F)	240/381	291/554	0.099
Age (y)	65.91±7.07	61.75±11.3	0.000
BMI (kg/m^2^)	25.11±3.43	26.53±3.77	0.000
WHR	0.87±0.06	0.89±0.31	0.041
FPG (mmol/L)	5.26±0.42	5.72±0.57	0.000
2hPPG (mmol/L)	6.01±1.07	8.57±1.33	0.000
TC (mmol/L)	5.24±1.00	5.28±1.11	0.523
TG (mmol/L)	1.54±0.99	1.86±1.28	0.000
HDL-C (mmol/L)	1.35±0.33	1.29±0.35	0.005
LDL-C (mmol/L)	3.23±0.94	3.25±1.50	0.732

p-DM, pre-diabetes;BMI, body mass index; WHR, waist-to-hip ratio; FBG, fasting blood glucose; 2hPPG, post OGTT 2 hours blood glucose; TC, total cholesterol; TG, triglycerides; HDL-C, high-density lipoprotein; LDL-C, low-density lipoprotein.

### Genotyping

Genomic DNA was extracted from 2–4 ml of peripheral blood using the salting-out method [20]. Six haplotype-tagging SNPs (*r* ^2^<0.8, MAF≥0.05) in the ATF6 gene (16 exons spanning 193 kb on chromosome 1q21–23) were selected from CHB data (Han Chinese in Beijing) in HapMap Phase II (R#23, http://www.hapmap.org). They were rs10918270 (G>A), rs12079579 (G>A), rs2134697 (G>A), rs2340721 (A>C), rs4657103 (G>T), and rs7522210 (G>C). The genotypes of all the 6 SNPs were determined by the MassArray system (Sequenom iPLEXassay, San Diego, United States) [21].

### Statistical analysis

All statistical analyses were performed using the SPSS statistical package (version 13.0; SPSS, Chicago, IL). Genotype distributions were tested at each polymorphic locus for departure from Hardy-Weinberg equilibrium. The clinical and laboratory values were expressed as means ± standard deviation (SD) or as medians (lower–upper quartiles). Comparisons of the clinical and laboratory parameters between the pre-diabetes and control groups, as well as between the genotypic groups, were performed with unpaired Student's *t*-tests or χ^2^ analysis, as appropriate. As a descriptive measure of the association between genotypes and pre-diabetes, odds ratios (ORs) were calculated along with 95% confidence intervals (CIs). Multifactor model analysis was used to assess hybrid effect. Pairwise LD was estimated using the combined data (cases and controls) by calculating |*D*′| and *r* ^2^ using Haploview (version 4.1) (http://www.broadinstitute.org/haploview/haploview) [13]. Haplotype block structure was determined using a CI algorithm [14] and haplotype frequencies were estimated with an expectation-maximization algorithm [15] using Haploview (v4.1). Bonferroni correction was used to correct for multiple comparisons.

## Results

Genotype distributions were in Hardy-Weinberg equilibrium at all six loci (*p*>0.01). Pre-diabetics were younger, and had higher BMI and waist-hip ratio (WHR), higher TG levels and lower HDL-C levels than control individuals (Table 1).

Carriers of genotype AA at rs2340721 had a higher risk for pre-diabetes than non-carriers (OR = 1.309, *p* = 0.013, β = 0.263). Using a multifactor model analysis to adjust for gender, age, BMI and WHR, the association of rs2340721 with pre-diabetes was significant (OR′ = 1.341, *p*′ = 0.011, β = 0.308). At rs7522210, GG homozygotes had a higher risk for pre-diabetes compared to non-carriers (OR = 1.390, *p* = 0.013, β = 0.316, adjusted OR′ = 1.516, *p*′ = 0.006, β = 0.366) (Table 2).

**Table 2:** Association between pre-diabetes and SNPs at the ATF6 locus.

SNPs	controls	p-DM						
/Genotypes	(n = 621)	(n = 845)	OR	95% CI	*p-value*	OR′	95% CI′	p′
rs10918270								
GG	343	447	0.955	0.775–1.177	0.666	1.006	0.807–1.254	0.956
AX	277	378	1					
rs12079579								
GG	396	515	0.940	0.757–1.166	0.573	0.951	0.762–1.186	0.653
AX	224	310	1					
rs2134697								
GG	396	515	1.039	0.734–1.470	0.380	1.000	0.693–1.443	0.999
AX	224	310	1					
rs2340721								
AA	222	347	1.309	1.050–1.613	0.013	1.341	1.063–1.669	0.011
CX	398	478	1					
rs4657103								
TT	491	677	1.202	0.924–1.564	0.171	1.177	0.891–1.554	0.251
GX	129	148	1					
rs7522210								
GG	88	152	1.390	1.206–1.818	0.013	1.516	1.101–2.006	0.006
CX	532	673	1					

SNPs,single nucleotide polymorphisms; p-DM, pre-diabetes.

OR′: adjusted for gender, age, BMI, and WHR.

*p*′: adjusted for gender, age, BMI, and WHR.

We also analyzed the interaction between environmental factors and SNPs on the risk of pre-diabetes. When BMI is treated as a continuous variable, there is a significant interaction with rs2340721 on pre-diabetic risk (*p* for the interaction = 0.003, β = 0.106).

The SNPs were distributed in two Linkage disequilibrium (LD) blocks. We also compared the haplotype distributions between pre-diabetes and controls. In block 1, haplotype TTAG (composed of rs4657103, rs2134697, rs2340721, and rs12079579) was found associated with pre-diabetes (*p* = 0.009) (Table 3).In block 2,haplotype (composed of rs10918270 and rs7522210) distributions between pre-diabetes and controls showed no statistical significant difference.

**Table 3:** Association analyses of haplotypes at the ATF6 locus with pre-diabetes.

	Haplotype frequencies		
Haplotype	p-DM	Controls	*p*-value
Block 1 (rs4657103-rs2134697-rs2340721-rs12079579)			
TTCG	0.556	0.643	0.066
TTAG	0.493	0.399	0.009
TCAA	0.260	0.253	0.765
GCAG	0.100	0.120	0.123
TCAG	0.012	0.013	0.877
Block2 (rs10918270-rs7522210)			
GG	0.752	0.673	0.143
GC	0.446	0.515	0.074
AC	0.356	0.348	0.800

p-DM, pre-diabetes.

*P*-value: comparison between pre-diabetes and control.

Of the 1,448 individuals, 748 had plasma insulin measured following 75 g OGTT. GG homozygotes at rs7522210 had higher FBG (GG vs. CX: 5.6±0.52 vs. 5.5±0.57 mmol/L, *p* = 0.016), lower HOMA-β (GG vs. CX: 77.0 vs. 79.6%, *p* = 0.004) and HOMA-IR levels (GG vs. CX: 1.79 vs. 1.84, *p* = 0.011), lower insulin levels (0, 30, and 120 minutes after glucose load, *p*<0.05), reduced AUCi (GG vs. CX: 67.3 [44.2–102.3] vs. 73.1 [49.4–111.4], *p* = 0.014) than non-carriers mainly in individuals with pre-diabetes (Table 4).

**Table 4:** β-cell function and insulin resistance for genotype groups at rs7522210 in pre-diabetes individuals.

	GG	CX	
	(n = 121)	(n = 627)	*P*
FBG (mmol/L)*	5.78±0.54	5.74±0.58	0.365
PBG30′ (mmol/L)*	10.08±1.95	10.06±1.73	0.928
PBG-2h (mmol/L)*	8.53±1.31	8.68±1.25	0.187
INS 0′#	8.1 (5.06–8.69)	9.8 (6.4–11.9)	0.001
INS 30′#	44.7 (25.7–52.6)	55.6 (27.3–70.8)	0.037
INS 120′#	59.8 (32.6–73.1)	79.3 (38.8–90.2)	0.031
HOMA-β#	72.7 (46.1–85.9)	90.3 (56.5–105.9)	0.107
HOMA-IR#	2.1 (1.26–2.36)	2.53 (1.61–3.13)	0.002
AUCi#	78.6 (50.5–101.5)	100.1 (54.3–117.7)	0.013
INS 30′/PBG30	21.90 (4.48–12.64)	13.10 (4.69–14.80)	0.336
INS 120′/PBG-2h	33.59 (10.66–33.20)	39.67 (10.97–38.56)	0.528

*data shown as mean ± SD.

# data shown as medians (lower–upper quartiles).

*P*′: adjusted for age, sex, and BMI.

BMI, body mass index; FBG, fasting blood glucose; PBG, post-OGTT glucose; INS0′#, fasting insulin levels; INS30′#, post-OGTT30′ insulin levels; INS120′#, 2 hours post-OGTT insulin levels;HOMA-β#, β-cell function; HOMA-IR#, homeostasis model assessments of insulin resistance; AUCi#, area under the curve for insulin;INS30′/PBG30, the ratio of INS30′ and PBG30; INS120′/PBG-2h, the ratio of INS120′ and PBG-2h.

We also found that the carriers of the GG genotype at rs10918270 had higher FBG (GG vs. AX: 5.568±0.548 vs. 5.501±0.580 mmol/L, *p* = 0.023), and rs4657103 was associated with PBG after 75 g glucose load (TT vs. GX: 7.590±1.726 vs. 7.316±1.785 mmol/L, *p* = 0.023).

## Discussion

The ER is a complicated membranous network in eukaryotic cells, and is the site for synthesis, folding, assembly, and posttranslational modification of proteins. It includes a highly conserved system of proteins that facilitates protein folding and processing, and protects cells from the toxic effects of accumulating unfolded proteins (i.e., ER stress). When these measures fail, it initiates apoptosis. This system, known as the UPR, is activated by the accumulation of unfolded proteins [3]–[5]. The UPR pathway is particularly important in secretory cells, including pancreatic β-cells, hepatocytes and adipocytes [3]. Many studies have demonstrated that UPR (including ATF6) is related to pancreatic β-cell function [16], and ATF6α may be important for maintaining β-cell survival [17], [18].

ATF6 is a key proximal sensor in the UPR pathway and is transported to the Golgi complex, where it is cleaved by proteases to yield an active cytoplasmic domain that upregulates chaperone proteins. It activates the UPR by regulating a group of genes encoding molecular chaperones and folding enzymes [6]. Its encoding gene includes 16 exons and spans 193 kb on chromosome 1q21–23, a region that is linked to type 2 diabetes in eight different populations [7], [8].

In this study, subjects with pre-diabetes were observed to determine the genetic factors that contribute to the very early stage of pancreatic β-cell dysfunction and thus play a role in the progression of type 2 diabetes.

Previous studies identified several SNPs in ATF6 associated with type 2 diabetes in different populations. In Pima Indians, rs2070150 (P145A) and rs1135983 (S157P) are associated with type 2 diabetes and reduced insulin response to oral glucose [12]; in Caucasians and African Americans, rs4579731, rs3820449 and rs10918215 are also associated with these traits [6], [9]–[12].

Two of the six haplotype-tagging SNPs selected for this study, rs2340721 and rs7522210, were respectively in high LD with two previously reported SNPs rs2070150 and rs1135983, in Caucasians (*r* ^2^ = 1) and CHB (*r* ^2^ = 1.0) in HapMap database. Also, the selected SNP rs4657103 was in high LD with the reported SNP rs4579731 in CHB (*r* ^2^ = 1.0). The SNPs linked to those found in other populations may be associated with Chinese pre-diabetes.

We found that rs2340721 was, indeed, associated with pre-diabetes. The carriers of genotype AA had a higher risk than non-carriers, even after adjusting for gender, age, BMI, and WHR. Furthermore, when BMI was treated as a continuous variable, it had an interaction effect with the locus on pre-diabetic risk.

The haplotype TTAG, composed of 4 SNPs including rs2340721 and rs4657103, was associated with pre-diabetes. rs7522210 was associated with FBG, insulin levels (0, 30, and 120 minutes after glucose load), AUCi, and HOMA-IR; rs10918270 was associated with FBG and rs4657103 with post-75 g glucose load (2 hour) glucose levels. These data suggest that genetic variation in ATF6 is associated with pre-diabetes and regulates pancreatic β-cell function in the population. BMI had a synergistic effect with genetic variation on the risk of pre-diabetes, which can be interpreted thatin clinical practice, individuals at a high risk of pre-diabetes will benefit from losing body weight; with the risk reduced by 10% per kg/m^2^ reduction in BMI. To date, no studies on the ATF6 gene specifically consider individuals with pre-diabetes.

In 2011, Hu et al. [19] reported no association between genetic variations in ATF6 and type 2 diabetes and its traits in a Chinese population. However, our data suggests that the genetic variants in ATF6 are associated with pre-diabetes, the early stage of type 2 diabetes,and its related traits. Firstly, the control subjects in our study were strictly screened, especially by age at 55 years or over. The high quality of control samples seem to be optimal for the studies on genetic factors. Secondly, some of the SNPs selected in the study were different from Hu's study, with SNPs rs2340721 and rs7522210 in common and none shared in the haplotype patterns. Thirdly, the individuals in our study were northern Chinese Han descent, living in Beijing, while eastern Chinese Hans in Hu's. Finally, we studied the subjects with pre-diabetes, rather than type 2 diabetes. This difference may indicate that the genetic variations in ATF6 play a more important role in the pre-diabetic stage than in the diabetic stage in the Chinese population, or likely there exists genetic heterogeneity within Chinese Han race. Additional studies are needed to confirm the hypothesis in different populations.

However, our study has some limitations. The small sample size might bias the results. Also, further functional studies are necessary. Our findings should be confirmed in prospective studies before ATF6 polymorphisms can be used to predict the risk of pre-diabetes in the Chinese population.

## Conclusion

The genetic variation in the ATF6 gene is associated with pre-diabetes and has interactive effects with BMI on pre-diabetes in the Chinese Han population.
